# Bicarbonate Evokes Reciprocal Changes in Intracellular Cyclic di-GMP and Cyclic AMP Levels in *Pseudomonas aeruginosa*

**DOI:** 10.3390/biology10060519

**Published:** 2021-06-10

**Authors:** Kasidid Ruksakiet, Balázs Stercz, Gergő Tóth, Pongsiri Jaikumpun, Ilona Gróf, Roland Tengölics, Zsolt M. Lohinai, Péter Horváth, Mária A. Deli, Martin C. Steward, Orsolya Dobay, Ákos Zsembery

**Affiliations:** 1 Department of Oral Biology, Semmelweis University, H-1089 Budapest, Hungary; ksd13rsk@gmail.com (K.R.); pongsirij@nu.ac.th (P.J.); martin.steward@me.com (M.C.S.); 2 Department of Conservative Dentistry, Semmelweis University, H-1085 Budapest, Hungary; lohinai.zsolt@dent.semmelweis-univ.hu; 3 Institute of Medical Microbiology, Faculty of Medicine, Semmelweis University, H-1089 Budapest, Hungary; stercz.balazs@med.semmelweis-univ.hu (B.S.); dobay.orsolya@med.semmelweis-univ.hu (O.D.); 4 Department of Pharmaceutical Chemistry, Semmelweis University, H-1092 Budapest, Hungary; toth.gergo@pharma.semmelweis-univ.hu (G.T.); horvath.peter@pharma.semmelweis-univ.hu (P.H.); 5 Institute of Biophysics, Biological Research Centre, H-6726 Szeged, Hungary; grof.ilona@brc.hu (I.G.); deli.maria@brc.hu (M.A.D.); 6 Institute of Biochemistry, Biological Research Centre, H-6726 Szeged, Hungary; tengolics.roland@brc.hu; 7 HCEMM-BRC Metabolic Systems Biology Laboratory, H-6726 Szeged, Hungary; 8 School of Medical Sciences, University of Manchester, Manchester M13 9PL, UK

**Keywords:** c-di-GMP, cAMP, *P. aeruginosa*, bicarbonate, pH, biofilm, cystic fibrosis, chronic infection

## Abstract

**Simple Summary:**

Cystic fibrosis (CF) is the most common lethal hereditary disease in Caucasians, causing mainly respiratory and gastrointestinal symptoms. In CF, mutations in the gene encoding an epithelial anion channel cause impaired bicarbonate secretion, which contributes to the formation of thick mucus in the airways. Together with reduced mucociliary clearance, this habitat is ideal for bacterial growth. Biofilm formation contributes to persistent infections and inflammation, as well as higher resistance to antibiotics, and therefore represents one of the major challenges for CF therapy. It is also known that second messenger molecules play pivotal roles in the regulation of bacterial lifestyle. Furthermore, the activities of the enzymes that synthesize or break down these second messengers are sensitive to external pH and bicarbonate. Therefore, we hypothesized that pH and/or bicarbonate could influence bacterial behavior. In this work, we have shown that bicarbonate per se regulates the concentrations of bacterial second messengers and inhibits biofilm formation. These results suggest that bicarbonate could be used as a supportive treatment in CF and possibly in other respiratory diseases associated with chronic bacterial infections and viscous mucus production.

**Abstract:**

The formation of *Pseudomonas aeruginosa* biofilms in cystic fibrosis (CF) is one of the most common causes of morbidity and mortality in CF patients. Cyclic di-GMP and cyclic AMP are second messengers regulating the bacterial lifestyle transition in response to environmental signals. We aimed to investigate the effects of extracellular pH and bicarbonate on intracellular c-di-GMP and cAMP levels, and on biofilm formation. *P. aeruginosa* was inoculated in a brain–heart infusion medium supplemented with 25 and 50 mM NaCl in ambient air (pH adjusted to 7.4 and 7.7 respectively), or with 25 and 50 mM NaHCO_3_ in 5% CO_2_ (pH 7.4 and 7.7). After 16 h incubation, c-di-GMP and cAMP were extracted and their concentrations determined. Biofilm formation was investigated using an xCelligence real-time cell analyzer and by crystal violet assay. Our results show that HCO**_3_**^−^ exposure decreased c-di-GMP and increased cAMP levels in a dose-dependent manner. Biofilm formation was also reduced after 48 h exposure to HCO**_3_**^−^. The reciprocal changes in second messenger concentrations were not influenced by changes in medium pH or osmolality. These findings indicate that HCO**_3_**^−^ *per se* modulates the levels of c-di-GMP and cAMP, thereby inhibiting biofilm formation and promoting the planktonic lifestyle of the bacteria.

## 1. Introduction

*Pseudomonas aeruginosa* is one of the most prevalent pathogens causing nosocomial infections in several organs, particularly chronic respiratory diseases [1]. Its pathogenicity is associated with biofilm formation, expression of various virulence factors, such as type II and III secretion systems (T2SS and T3SS) and type IV pili, and reduced susceptibility to antibiotics, all of which largely depend on altered intracellular second messenger levels [2,3,4,5]. Bis-(3′-5′)-cyclic dimeric guanosine monophosphate (c-di-GMP) is a bacterial second messenger that mediates the lifestyle transition from motility to sessility [2,6]. In *P. aeruginosa*, c-di-GMP also regulates other biological processes, including virulence, quorum sensing, and cross-talk with other second messengers such as 3′,5′-cyclic adenosine monophosphate (cAMP) and hyperphosphorylated guanosine derivatives (p)ppGpp [4,7]. Intracellular c-di-GMP levels are elevated by activation of diguanylate cyclases (DGCs) and reduced by stimulation of c-di-GMP phosphodiesterases (PDEs) [2,6]. Cyclic AMP was first described in eukaryotic cells but subsequently found also in bacteria [3]. It is responsible for modulating catabolic metabolism, virulence factor expression, and lifestyle transitions. Intracellular cAMP levels are regulated by the concerted action of adenylate cyclases (ACs) such as CyaA, CyaB or ExoY, and cAMP PDEs [3]. The activity of enzymes regulating c-di-GMP and cAMP concentrations can be influenced by extracellular signaling molecules, which may act at transcriptional, translational and/or post-translational levels [2,3]. For example, nitric oxide (NO) induces biofilm dispersion by altering c-di-GMP PDE activity [8]. Furthermore, as a host-activated AC toxin, cytosolic Ca^2+^ concentrations can determine T3SS activity by controlling cAMP concentration [3]. Our previous work also indicates that alkaline pH and HCO_3_^−^ increase intracellular cAMP levels and reduce biofilm formation [9].

Bicarbonate ions possess antibacterial properties that enhance the efficacy of both antimicrobial peptides [10] and synthetic antibiotics [11]. It has also been shown that NaHCO_3_ can disrupt mature dental biofilms [12]. Recently, we have reported that HCO_3_^−^ is bacteriostatic in media modified to mimic cystic fibrosis (CF) sputum [13]. HCO_3_^−^ is useful not only in medicine, but also in industry as a disinfectant [14] or antiviral agent [15]. In addition, HCO_3_^−^ plays a pivotal role in epithelial fluid secretion and calcium chelation, determining the tertiary structure of secreted mucins [16] in several organs such as the airways, gastrointestinal, and reproductive tracts [17,18]. HCO_3_^−^ secretion is impaired in CF due to mutations in the gene encoding the cystic fibrosis transmembrane conductance regulator (CFTR) protein [16]. Hyposecretion of HCO_3_^−^, in parallel with hyperabsorption of Na^+^, leads to airway surface liquid (ASL) dehydration. As HCO_3_^−^ typically maintains pH balance in the airways, defective CFTR function also causes increased acidity of the ASL and it eventually weakens immune-cell function. Due to low HCO_3_^−^ levels, a thick mucus is accumulated, leading to airway blockage and decreased mucociliary clearance (MCC) [19,20]. These conditions provide a favorable habitat for bacterial colonization [20].

*P. aeruginosa* is one of the most common bacteria colonizing the adult CF lung. In chronic infections, *P. aeruginosa* forms a biofilm enclosed in a self-produced extracellular matrix which provides protection against the host’s immune response and against natural and synthetic antibiotics, leading to the high morbidity and mortality of CF patients [21]. Given the potential therapeutic value of HCO_3_^−^, it is encouraging to note that Gomez et al., have demonstrated that administration of hypertonic NaHCO_3_ aerosols is safe and well-tolerated by CF patients [22].

Interplay between c-di-GMP and cAMP has been shown to control the lifestyle transition and virulence of *P. aeruginosa* [5,23]. Therefore, targeting these second messengers might be an effective therapeutic approach to combat bacterial colonization in CF airways. Since many small extracellular molecules, such as glycosylated triterpenoid saponin, sodium orthovanadate, and phosphoserine, as well as HCO_3_^−^/CO_2_, are known to modulate enzymes associated with second messenger production and degradation [24,25,26,27], we hypothesized that extracellular HCO_3_^−^ could also modulate the cAMP and/or c-di-GMP levels influencing biofilm formation. Therefore, our aim was to investigate the effects of HCO_3_^−^ on second messengers and biofilm formation in *P. aeruginosa*. Data presented here indicate that HCO_3_^−^ causes reciprocal changes in cAMP and c-di-GMP levels as well as inhibiting biofilm formation.

## 2. Materials and Methods

### 2.1. Growth Conditions and Bacterial Strains

Brain–heart infusion (BHI) medium (Mast Group Ltd., Merseyside, UK) was supplemented with either NaHCO_3_ or NaCl as follows: 1) BHI alone pH 7.4; 2) BHI + 25 mM NaCl pH 7.4; 3) BHI + 25 mM NaHCO_3_ pH 7.4; 4) BHI + 50 mM NaCl, pH 7.7; and 5) BHI + 50 mM NaHCO_3_ pH 7.7. The pH of BHI and BHI media supplemented with NaCl was adjusted with NaOH or HCl, whereas BHI medium supplemented with NaHCO_3_ was equilibrated with 5% CO_2_ to obtain the pH values indicated above. Single colonies of *P. aeruginosa*, ATCC 27853 and 17808 (clinical isolate), were cultured overnight.

The optical density at 595 nm (OD_595_) of the overnight cultures was measured with a PR2100 microplate reader (Bio-Rad Laboratories, Hercules, CA, USA) and the cultures were then diluted to OD_595_ = 0.4. They were inoculated in triplicate in 20 mL of designated medium for each condition. Cultures in BHI medium with added NaCl were incubated at 37 °C in ambient air, while the NaHCO_3_ groups were incubated in the presence of 5% CO_2_.

### 2.2. Extraction of c-di-GMP and cAMP

The extraction method was modified from that used by Petrova and Sauer (2017) [28]. Briefly, after 16 h incubation, the OD_595_ values of the bacterial cultures were determined. To obtain the same number of bacteria for extraction from each culture, a sample volume was calculated, equivalent to 20 mL of OD_595_ = 0.2. For example, if the OD_595_ was 0.4, the sample volume would be 10 mL. Bacterial cells were harvested by centrifugation at 5000× *g* rpm for 10 min, and the media were discarded. They were then washed three times with 1 mL ice-cold PBS. The cell pellets were resuspended in 100 μL ice-cold PBS and the extraction of cyclic nucleotides was initiated by incubation at 100 °C for 5 min, followed by the addition of 186 μL ice-cold absolute ethanol. The resulting solution was centrifuged and the supernatant, containing the extracted cyclic nucleotides, was removed and kept on ice. The extraction with heat and ethanol was repeated twice from the retained cell pellets. The supernatants from the three extractions were then pooled together in one tube and dried in a centrifugal evaporator (Labconco Centrivap Concentrator, Kansas City, MO, USA). The remaining cell pellets were kept for protein measurement. They were resuspended in 1 mL of 0.1 M NaOH, incubated at 95 °C for 15 min, and then sonicated on ice as previously described [29]. The protein measurement was carried out using a Quant-iT™ protein assay (Thermo Fisher Scientific, Waltham, MA, USA), according to the manufacturer’s instructions. Finally, total cellular protein content was used to normalize the c-di-GMP and cAMP levels obtained from the quantification. Three independent bacterial cultures were performed in all cases.

### 2.3. Quantification of c-di-GMP and cAMP

The extracted c-di-GMP and cAMP were detected by high-performance liquid chromatography with mass spectrometric detection (HPLC-MS) according to a modified protocol from the above-mentioned study [28]. HPLC analysis was performed using an Agilent 1260 Infinity LC system in conjunction with an Agilent 6460 triple-quadrupole mass spectrometer (Waldbroon, Germany). Chromatography was carried out using an Agilent Eclipse Plus C18 column (4.6 × 100 mm, 3.5 μm). For the separation, the following gradient elution program was used, mixing Solvent A (10 mM ammonium acetate in water) and solvent B (10 mM ammonium acetate in methanol): 0 to 9 min 1% B, 9 to 14 min 15% B, 14 to 19 min 25% B, 19 to 26 min 90% B and 26 to 30 min 1% B. The flow rate was 0.3 mL/min. Standards for c-di-GMP and cAMP quantification were purchased from Sigma Aldrich (St. Louis, MO, USA). The mass spectrometer was operated in conjunction with a Jet Stream electrospray ion source in positive ion mode and was set to monitor in selective ion monitoring (SIM) mode. The following MS parameters were used: fragmentor voltage 130 V; dwell time 200 ms; delta EMV 10 V. Flow and temperature of the drying gas (N_2_) in the ion source were 10 L/min and 300 °C, respectively; the pressure of the nebulizer gas (N_2_), 45 psi; capillary voltage, 3000 V; sheath gas flow and temperature, 10 L/min and 300 °C. Mass spectra were processed using Agilent MassHunter B.04.00 software. The dried supernatant samples from the bacterial extracts were resolved in nanopure water, then vortexed and centrifuged to remove insoluble particles. The samples were carefully filtered with a 2 µm filter into a new microcentrifuge tube. We analyzed the final volume (20 µL per sample) using HPLC-MS.

### 2.4. Real-Time Biofilm Monitoring

For the biofilm experiments, BHI medium was supplemented with 1% glucose and sterilized by filtration. BHI medium supplemented with either 25 or 50 mM NaHCO_3_ was incubated at 5% CO_2_ and prepared as described above. The pH of media supplemented with NaCl (25 or 50 mM) was adjusted with NaOH to pH 8.0 or pH 8.4, respectively. We set the pH at 0.6–0.7 units higher than the desired values because the experiments were carried out in the presence of 5% CO_2_, which reduced the pH during the measurements. Similarly, the pH of BHI medium alone was adjusted to either 8.0 or 8.4 and used as the control medium.

96-well E-plates were used in conjunction with a real-time cell analyzer (RTCA) (xCELLigence, ACEA Bioscience Inc., San Diego, CA, USA). After overnight culture, the ODs were determined and standardized to 10^9^ CFU/mL by dilution with BHI medium at pH 7.4 containing 1% glucose. Bacterial suspensions were obtained, equivalent to 20 µL of 10^8^ CFU/mL, and inoculated in 180 µL of the designated medium, resulting in final volumes of 200 µL per well. Five replicate wells were prepared for each condition. The E-plate was incubated at 37 °C with 5% CO_2_ for 48 h. After bacterial inoculation, the RTCA impedance signal was recorded every 10 min for 48 h. The recorded signals obtained at 6, 12, 24 and 48 h were converted by the xCELLigence software to delta cell indices (∆CI).

### 2.5. Biofilm Assessment Using Crystal Violet Assay

*P. aeruginosa*, both ATCC 27853 and 17808 (clinical isolate), were grown in 96-well polystyrene flat-bottom microtiter plates (Eppendorf^®^, 0030730119, Hamburg, Germany). BHI media supplemented with 25 and 50 mM NaHCO_3_ were prepared as described above. The pH values of the BHI media supplemented with NaCl were adjusted in the same way as for the impedance-based RTCA method. Briefly, 20 µL samples of the overnight cultures diluted to OD_595_ = 0.1 were inoculated in wells containing 180 μL designated medium in 5 parallels for each group. Sterile distilled water (200 μL) was added to each empty well as previously recommended [30,31]. The plate was incubated for 48 h at 37 °C in 5% CO_2_. 180 μL of the supernatant was carefully aspirated from each well, which was then washed by adding 200 μL PBS and discarding the supernatant. This washing step was repeated twice. The plate was then dried at 42.5 °C for 90 min. 200 μL of 0.1% crystal violet was added to each well to stain the biofilm. After 15 min, the excess crystal violet was removed and the well washed with distilled water. The plate was dried at room temperature for 30 min. Finally, 200 μL of 30% acetic acid was added to each well for 15 min, and 125 μL of the dissolved crystal violet was transferred to a new plate for OD_595_ measurement. Three independent biological cultures were performed.

### 2.6. Statistical Analysis

Normalized c-di-GMP and cAMP concentrations were calculated by Microsoft Excel for Office 365 using a previously described formula [28]. Data are presented as means ± standard error (SEM). One-way ANOVA was used to analyze the second-messenger results, whereas a two-way ANOVA, followed by a multiple comparison test, was used for the biofilm results. GraphPad Prism version 8.0.0 was used for statistical analysis. Significance was accepted at *p* < 0.05.

## 3. Results

### 3.1. Sodium Bicarbonate Modulates Both c-di-GMP and cAMP Levels in P. aeruginosa

#### 3.1.1. Sodium Bicarbonate Decreases Intracellular c-di-GMP Levels

**Figure 1:**
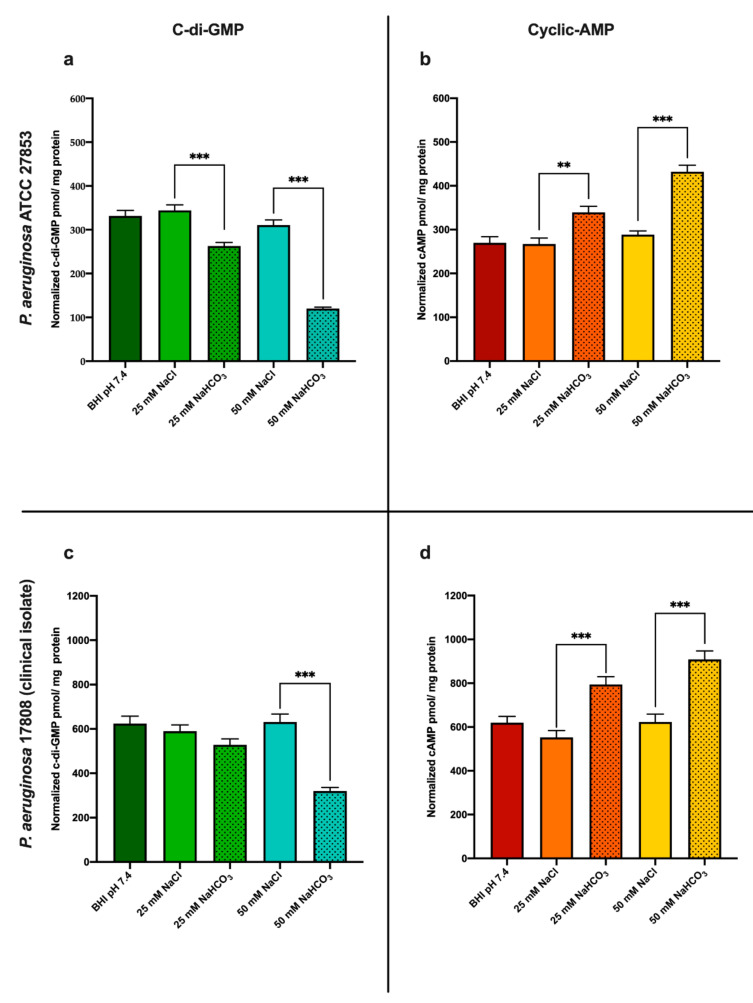
Intracellular second messenger levels after 16 h incubation: (**a**) c-di-GMP levels and (**b**) cAMP levels in *P. aeruginosa* ATCC 27853; (**c**) c-di-GMP levels, and (**d**) cAMP levels in *P. aeruginosa* 17808 (clinical isolate). Values are presented as means ± SEM of the c-di-GMP or cAMP levels normalized to cellular protein from 3 independent experiments. One-way ANOVA and Tukey’s multiple comparisons test: ** *p* < 0.01 and *** *p* < 0.001 when comparing cells in BHI medium supplemented with NaHCO_3_ and cells in BHI medium supplemented with an equal concentration of NaCl at the same pH.

In order to test the effects of HCO_3_^−^ on intracellular c-di-GMP levels in *P. aeruginosa* (ATCC 27853 and clinical isolate 17808), we incubated the bacteria for 16 h in BHI medium supplemented with either NaHCO_3_ (25 or 50 mM) or NaCl (25 or 50 mM). In *P. aeruginosa* ATCC 27853, c-di-GMP levels were significantly reduced in media containing both 25 and 50 mM NaHCO_3_ compared to media containing 25 and 50 mM NaCl, respectively (Figure 1a). In *P. aeruginosa* clinical isolate 17808, 50 mM NaHCO_3_ decreased c-di-GMP concentrations significantly, but 25 mM did not (Figure 1c). These data show that the NaHCO_3_-induced decreases in c-di-GMP levels were dose-dependent in both *P. aeruginosa* ATCC 27853 and clinical isolate 17,808 (Figure 1a,c). Neither 25 mM NaCl (pH 7.4) nor 50 mM NaCl (pH 7.7) induced changes in c-di-GMP concentrations, suggesting that alterations in pH or osmolarity did not play a role in these inhibitory effects. Thus, our results indicate that it is HCO_3_^−^ *per se* that decreases the intracellular c-di-GMP concentrations.

#### 3.1.2. Sodium Bicarbonate Increases Intracellular cAMP Levels

In parallel with the c-di-GMP measurements, we also investigated the changes in intracellular cAMP concentration induced by NaHCO_3_. As shown in Figure 1b (*P. aeruginosa* ATCC 27853) and Figure 1d (*P. aeruginosa* clinical isolate 17808), both 25 and 50 mM NaHCO_3_ elevated cAMP levels when compared to treatments with equimolar concentrations of NaCl at the same pH values. Supplementation of BHI medium with 25 or 50 mM NaCl caused no significant change in cAMP concentration, suggesting that the HCO_3_^−^-induced effects were not due to the accompanying osmolarity or pH changes (Figure 1b,d).

### 3.2. Sodium Bicarbonate Inhibits P. aeruginosa Biofilm Formation

#### 3.2.1. Effects of Sodium Bicarbonate Assessed by Real-Time Cell Analysis (RTCA)

**Figure 2:**
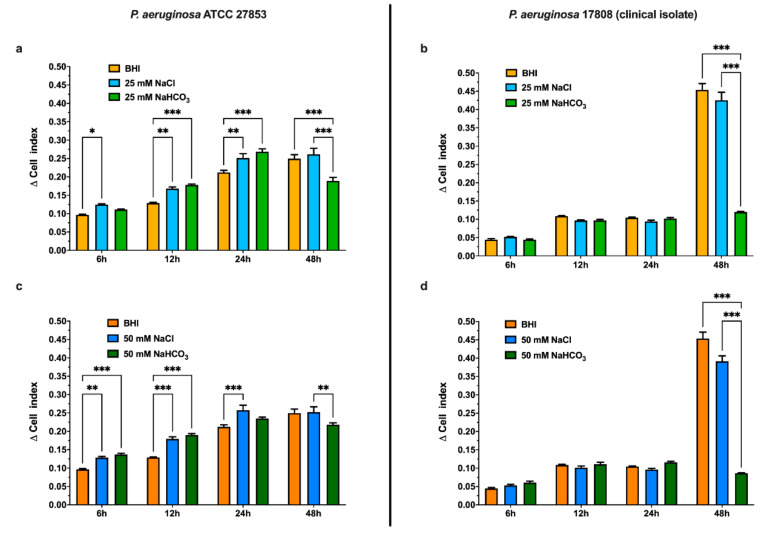
Biofilm formation by *P. aeruginosa* ATCC 27853 (**a**,**c**) and *P. aeruginosa* 17808 (clinical isolate) (**b**,**d**) in media supplemented with 25 mM (**a**,**b**) or 50 mM (**c**,**d**) NaCl or NaHCO_3_ using real-time biofilm monitoring (RTCA) at specified time points. Values are presented as means of Δ cell index ± SEM from 4–5 parallel measurements. Two-way ANOVA and Tukey’s multiple comparisons test: * *p* < 0.05, ** *p* < 0.01 and *** *p* < 0.001 when comparing pure BHI medium, BHI medium supplemented with NaHCO_3_, and BHI medium supplemented with an equal concentration of NaCl.

The ability of *P. aeruginosa* to form biofilms was assessed by RTCA and quantified as the delta cell index (ΔCI), a parameter, which increases with biofilm formation. Although biofilm formation generally requires at least 24 h, here we also present data following 6 and 12 h incubation. Interestingly, in the clinical isolate, ΔCI increased dramatically at 48 h, indicating particularly strong biofilm formation (Figure 2b).

The effects of HCO_3_^−^ were investigated in BHI medium supplemented with either NaHCO_3_ (25 or 50 mM) or NaCl (25 or 50 mM) as a control (Figure 2). Regardless of whether the BHI medium was supplemented with NaHCO_3_ or NaCl, ΔCI increased with incubation time in both the ATCC strain and the clinical isolate. As with the BHI medium alone, ΔCI increased dramatically in the clinical isolate incubated in BHI medium supplemented with 25 or 50 mM NaCl for 48 h (Figure 2b,d). However, both 25 and 50 mM NaHCO_3_ greatly reduced ΔCI, compared with the same concentration of NaCl, following 48 h incubation. This indicates an inhibition of biofilm formation that was particularly pronounced in the clinical isolate culture (Figure 2b,d).

#### 3.2.2. Effects of Sodium Bicarbonate Assessed by Crystal Violet Assay

**Figure 3:**
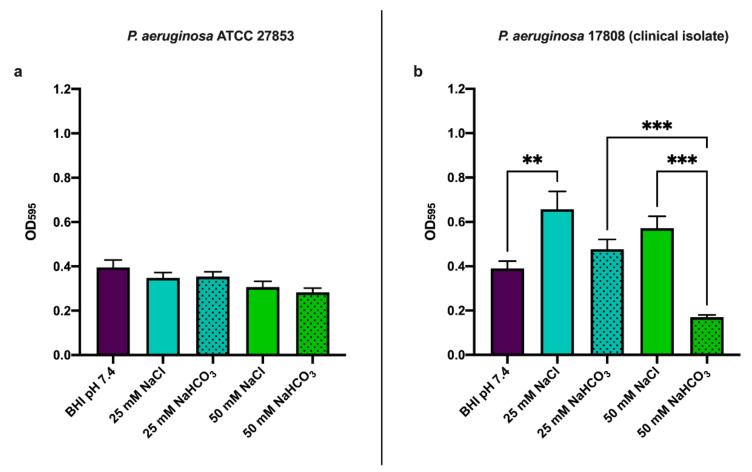
Biofilm formation at 48 h using crystal violet assay: (**a**) *P. aeruginosa* ATCC 27853 and (**b**) *P. aeruginosa* 17808 (clinical isolate). Values are presented as means of OD_595_ ± SEM from 3 independent experiments. One-way ANOVA and Tukey’s multiple comparisons test: ** *p* < 0.01 and *** *p* < 0.001 when comparing BHI medium supplemented with NaHCO_3_ and BHI medium supplemented with the same concentration of NaCl.

As a further test of the effects of NaHCO_3_ on biofilm formation, we used the crystal violet assay after 48 h incubation (Figure 3). In *P. aeruginosa* ATCC 27853, neither 25 mM nor 50 mM NaHCO_3_ decreased biofilm formation as judged by this assay (Figure 3a). However, 50 mM NaHCO_3_ did significantly reduce biofilm formation by *P. aeruginosa* 17808 (clinical isolate) (Figure 3b). Interestingly 25 mM NaCl increased *P. aeruginosa* 17808 (clinical isolate) biofilm formation (Figure 3b). These data also suggest that changes in osmolarity or pH are not responsible for the inhibitory effect of NaHCO_3_.

## 4. Discussion

Acid-base transporters regulate ASL pH, which is essential to the homeostasis of the respiratory system. CFTR plays a pivotal role in HCO_3_^−^ secretion across airway epithelia, and impaired HCO_3_^−^ secretion in CF leads to an acidic luminal pH that provides favorable conditions for *P. aeruginosa* colonization [16,21]. In *P. aeruginosa,* enzymes regulating the levels of intracellular second messengers (c-di-GMP and cAMP) are known to be sensitive to changes in environmental HCO_3_^−^ concentrations. Since both c-di-GMP and cAMP may influence biofilm formation, alterations in their concentrations could play an important role in chronic CF airway infections [2,3,21]. The main findings of the current study demonstrate that HCO_3_^−^ administration evokes reciprocal changes in c-di-GMP and cAMP concentrations in *P. aeruginosa*, which result in inhibition of biofilm formation.

Bacteria can exist either as free-floating planktonic cells or as sessile colonies forming biofilms. It has been demonstrated that high intracellular levels of c-di-GMP promote biofilm formation, whereas lower concentrations induce the planktonic lifestyle [2,6,32]. Our results indicate that both 25 and 50 mM NaHCO_3_ reduce c-di-GMP levels in the ATCC strain of *P. aeruginosa*. In the clinical isolate strain, 50 mM but not 25 mM NaHCO_3_ reduced c-di-GMP levels, suggesting a different sensitivity of these bacteria to external HCO_3_^−^ (Figure 1a,c).

In general, modulation of intracellular c-di-GMP concentrations may be explained either by activation of PDEs and/or inhibition of DGCs. For example, Koestler and Waters showed that, in *Vibrio cholerae*, HCO_3_^−^ and bile acids can suppress DGCs activity and simultaneously stimulate PDEs, both leading to decreased c-di-GMP levels [33]. Clearly, further studies are needed to identify the molecular pathways by which HCO_3_^−^ modulates c-di-GMP levels in *P. aeruginosa*.

*P. aeruginosa* isolates from CF airways and/or sputum are typically derived from the same strain but show extensive phenotypic heterogeneity [34]. Some of these variants develop an increased ability to form biofilms and are frequently resistant to antibiotics. These are called small colony variants (SCVs) [35]. The underlying mechanisms responsible for the generation of SCVs are still unclear, but the final common pathway seems to be an increase in bacterial c-di-GMP concentrations [36,37]. SCVs also produce large amounts of exopolysaccharides that play a crucial role in the development of antibiotic resistance [35,38]. Elevated c-di-GMP has been recognized as essential for promoting the SCV phenotype change and is thought to occur as a result of overexpression and/or activation of DGCs such as WspR or YfiN (TbpB) [2].

Our findings clearly support the idea that inhalation of NaHCO_3_-containing aerosols might influence the *P. aeruginosa* phenotype change in CF. Either by reducing DGC activity or increasing PDE activity, HCO_3_^−^ exposure could effectively lower bacterial c-di-GMP levels and reduce the likelihood of biofilm formation in the airways.

Aside from c-di-GMP-mediated biofilm formation, cAMP may also participate in this process. We therefore also investigated the effects of HCO_3_^−^ on bacterial cAMP levels. In contrast to the effects on c-di-GMP, HCO_3_^−^ increased cAMP concentrations in a dose-dependent manner. Our observations are in line with previous studies showing similar results in a range of 5 to 25 mM NaHCO_3_, which activated CyaB and increased intracellular cAMP levels [27]. Furthermore, HCO_3_^−^ can also stimulate soluble AC in both bacterial and mammalian cells—another pathway leading to increased intracellular cAMP concentrations [39,40]. Although we cannot exclude the possibility that PDE activities are also regulated by HCO_3_^−^, no current evidence is available to support this hypothesis.

By assessing changes in bacterial c-di-GMP and cAMP levels concurrently, we found that HCO_3_^−^ regulates c-di-GMP and cAMP levels in a reciprocal fashion. How the synthesis and degradation of these second messengers are coupled is not fully understood. It has been previously demonstrated that the accumulation of intracellular cAMP inhibits irreversible attachment, and consequently decreases biofilm formation in *P. aeruginosa* [23,41]. More recently, Almblad and colleagues have described a subset of c-di-GMP PDEs that are involved in the cAMP–Vfr regulated suppression of c-di-GMP concentrations [23]. Therefore, an increase in cAMP concentrations can itself decrease c-di-GMP levels in *P. aeruginosa*, which in turn also inhibits biofilm formation [5,23]. On the other hand, high levels of c-di-GMP can decrease cAMP concentrations and suppress numerous acute virulence factors such as T2SS and T3SS, and type IV pili, although the detailed mechanisms of this regulatory pathway are still unknown [5].

To investigate the effect of HCO_3_^−^ on biofilm formation in real-time, we applied an impedance-based detection method. In the field of microbiology, this approach is used to detect behavioral differences between biofilm and non-biofilm-producing strains [42], as well as to assess antiseptic [43] and antibiotic efficacy [44]. Data obtained by this method can be presented as either normalized cell index or Δ cell index [45]. Here we considered values of Δ cell index due to the noticeable drop in initial cell index (leading to negative ΔCI values) in the presence of HCO_3_^−^. It should also be kept in mind that we performed these experiments in the presence of 5% CO_2_ so that BHI medium alone or BHI medium supplemented with NaCl also contained small amounts of HCO_3_^−^ which could have influenced bacterial growth and biofilm formation, although there is no indication of this in the results.

The observed reduction in Δ cell index in the RTCA measurements indicates that NaHCO_3_ (25 and 50 mM) significantly decreases biofilm formation in both the ATCC strain and the clinical isolate at 48 h (Figure 2). It is also worth mentioning that Δ cell index gradually increased up to 24 h in the ATCC strain (Figure 2a,c), and the values were significantly higher in BHI medium supplemented with NaCl, and with NaHCO_3_, than in the control medium. These data suggest that increased ionic strength may also influence initial bacterial adhesion.

Biofilm formation by *P. aeruginosa* is commonly assessed by crystal violet assay following at least 24 h incubation. Using the crystal violet assay, we were able to detect the inhibitory effects of 50 mM NaHCO_3_ on biofilm formation in the clinical isolate but not in the ATCC strain (Figure 3a,b). Although we had observed similar changes in bicarbonate-induced cyclic nucleotide concentrations in the two strains, other factors might be involved in determining their biofilm-forming capacity. There are inconsistent data in the literature regarding the biofilm-forming capacity of *P. aeruginosa* ATCC 27853 [46,47]. In addition, *P. aeruginosa* generally forms biofilms at an air–liquid interface which can be defined as floating biofilms [48,49,50]. Under these conditions, the cells produce a viscous matrix containing large amounts of water. Therefore, the crystal violet assay may not be reliable for quantifying *P. aeruginosa* biofilm formation, as has been previously suggested [51].

In a previous study, we reported that bicarbonate increased intracellular cAMP levels and decreased biofilm formation in *P. aeruginosa* [9]. Since the ELISA-based method used in that study was not suitable for detecting changes in c-di-GMP and cAMP concomitantly, we have applied the highly sensitive HPLC-MS technique in the present study. Furthermore, we have also used an impedance-based approach to assess the effects of bicarbonate on biofilm formation. As a further extension of our previous observations, we have examined the dose-dependence of the effects of NaHCO_3_ by using both 25 and 50 mM concentrations.

Taken together, our findings indicate that HCO_3_^−^ *per se* decreases intracellular c-di-GMP in *P. aeruginosa* while increasing cAMP levels. These bicarbonate-induced reciprocal changes in second messenger concentrations inhibit biofilm formation. Although we show here that these effects of HCO_3_^−^ were independent of pH, the alkalization of the ASL that would accompany exogenous administration of HCO_3_^−^ would also have the beneficial effect of reducing the viscosity of CF sputum [52,53], thereby facilitating mucociliary clearance. Importantly, both in vitro experimental [54] and in vivo clinical data [22] indicate that the application of bicarbonate is safe in CF. In addition, nebulized sodium bicarbonate has no adverse effect on airway smooth muscle [55]. Therefore, we propose that aerosolized NaHCO_3_ could be very effective as a supportive treatment in CF, and possibly in other respiratory diseases associated with chronic bacterial infections and viscous mucus production, such as chronic obstructive pulmonary disease (COPD).

## Data Availability

Data sharing is not applicable to this article.
